# S111. ARE SCHIZOPHRENIA AND SCHIZO-AFFECTIVE DISORDER SEPARABLE?

**DOI:** 10.1093/schbul/sby018.898

**Published:** 2018-04-01

**Authors:** Walter Heinrichs, Leah Hartman, Farena Pinnock, Farzaneh Mashhadi, Sarah Ciantar

**Affiliations:** York University

## Abstract

**Background:**

Resolving the definition, heterogeneity and validity of schizophrenia-spectrum disorders remains a challenge, including the distinctiveness of schizophrenia and schizoaffective disorder. Here we report clinical, cognitive and structural brain imaging data with special reference to social processing in corresponding patient groups and non-psychiatric control participants. The study question was: to what extent do these data support schizophrenia and schizoaffective disorder as separable biobehavioural syndromes of psychotic illness?

**Methods:**

DSM-V criteria were applied to an outpatient sample, yielding n=44 with schizophrenia and n=29 with schizoaffective disorder. In addition to demographic data, symptom severity was measured in both patient groups with the Positive and Negative Syndrome Scale (PANSS). Overall cognition was measured with the MATRICS Consensus Cognitive Battery (MCCB) composite and social cognition with Theory of Mind, emotion perception and attribution bias tasks. Cortical thickness in regions associated with the social brain network was measured with a 3T General Electric MRI short bore scanner, with parcellations obtained using methods described by Destrieux et al. (2010) in Freesurfer. Non-psychiatric control participants (n=63) were studied with cognitive, social cognitive and MRI measures for comparison.

**Results:**

Study groups did not differ in age, educational achievement, proportion of males or prevalence of English as the preferred language. Patient groups did not differ in symptom severity (PANSS) or anti-psychotic medication (1st versus 2nd generation), but did differ significantly in terms of independent living, with schizoaffective patients significantly more independent than schizophrenia patients. The composite MCCB index and theory of mind task revealed significant differences between controls and patient groups, but no differences between patient groups. Schizophrenia patients differed significantly from both schizoaffective and control participants on the emotion perception task. There were no group differences in attribution bias. Multivariate analysis of variance (MANOVA) revealed that cortical thickness values in the social network were significantly lower in patient groups relative to controls for 14 regions. There were no schizophrenia vs schizoaffective group differences following correction. However, 9 regions were significantly reduced in schizophrenia patients relative to controls and 5 regions in schizoaffective patients relative to controls. Cingulate gyrus and superior temporal sulcus regional differences remained significant following correction.

**Discussion:**

Although schizophrenia and schizoaffective disorder continue to be recognized as distinct syndromes in some diagnostic systems (e.g. DMS V), the validity of the distinction remains in question. Apart from functional independence, which may in part be an artifact of the diagnostic criteria, and aspects of emotion perception, we found no evidence to support longstanding conjectures that these syndromes are distinct, at least not in terms of the clinical, cognitive, social cognitive and social brain network-associated measures used in this study.

